# Cellular and Molecular Pathophysiology of Traumatic Brain Injury: What Have We Learned So Far?

**DOI:** 10.3390/biology12081139

**Published:** 2023-08-17

**Authors:** Marco Aurelio M. Freire, Gabriel Sousa Rocha, Leonardo Oliveira Bittencourt, Daniel Falcao, Rafael Rodrigues Lima, Jose Rodolfo Lopes P. Cavalcanti

**Affiliations:** 1 Graduate Program in Physiological Sciences, University of the State of Rio Grande do Norte, Mossoró 59607-360, RN, Brazil; 2 Graduate Program in Biochemistry and Molecular Biology, University of the State of Rio Grande do Norte, Mossoró 59607-360, RN, Brazil; 3 Laboratory of Functional and Structural Biology, Institute of Biological Sciences, Federal University of Pará, Belém 66075-900, PA, Brazil; 4 VCU Health Systems, Virginia Commonwealth University, 23219 Richmond, VA, USA

**Keywords:** traumatic brain injury, brain edema, inflammation, excitotoxicity, oxidative stress

## Abstract

**Simple Summary:**

Traumatic brain injury (TBI) is a catastrophic event to the health and life of its sufferers, and it also affects their relatives. In addition to the underlying complications of the resulting condition of affected individuals, there are substantial implications for health and social security systems, since the productive capacity of people who suffer the consequences of a TBI is greatly limited. In this sense, it is essential that studies aimed at a better understanding of the physiological changes resulting from a TBI be carried out. Our work aims to review the main factors related to the alteration of injured brain tissue after TBI, such as inflammatory response, excitotoxicity, and oxidative stress. The adequate understanding of the phenomena resulting from TBI can help improve the care, rehabilitation process and quality of life of the affected people in addition to reducing the associated economic impact.

**Abstract:**

Traumatic brain injury (TBI) is one of the leading causes of long-lasting morbidity and mortality worldwide, being a devastating condition related to the impairment of the nervous system after an external traumatic event resulting in transitory or permanent functional disability, with a significant burden to the healthcare system. Harmful events underlying TBI can be classified into two sequential stages, primary and secondary, which are both associated with breakdown of the tissue homeostasis due to impairment of the blood–brain barrier, osmotic imbalance, inflammatory processes, oxidative stress, excitotoxicity, and apoptotic cell death, ultimately resulting in a loss of tissue functionality. The present study provides an updated review concerning the roles of brain edema, inflammation, excitotoxicity, and oxidative stress on brain changes resulting from a TBI. The proper characterization of the phenomena resulting from TBI can contribute to the improvement of care, rehabilitation and quality of life of the affected people.

## 1. Introduction

The brain is undoubtedly the most complex and intriguing structure in the human organism. Its organization is characterized by a myriad of cells with distinct morphophysiological characteristics that establish an intricate network of connections that originate and modulate all the individual’s behaviors. Though, despite the ever-increasing range of information about this organ, much remains to be determined, especially concerning tissue and cell responses as a result of disturbances of any nature, either mechanical (traumatic), chemical or physiological.

One of the most common and detrimental events that can generate permanent impacts on brain functioning is traumatic brain injury (TBI), which is a complex and heterogeneous disorder in the brain structure as a result of an external force in the form of mechanical, electrical, thermal or chemical energy, or a set of these, applied on it [1], emerging as a serious public health concern globally [2,3]. TBI corresponds to the third most prevalent cause of death and neurological impairment worldwide, also resulting in serious dysfunctions that severely interfere with the quality of life of affected individuals [4]. In the long term, a TBI can, through secondary damage, lead to neurodegenerative pathologies such as Alzheimer’s disease, Parkinson’s disease and dementia [5].

Projections from the Global Burden of Disease (GBD) point out that the incidence of TBI tends to increase in the coming years due to the growth in population density and the increasing use of automobiles, motorcycles and bicycles as a way of transport [6]. One of the most devastating consequences of a TBI is the cognitive and/or functional impairment observed in surviving patients, which is directly associated to the degree of the injury [3], generating not only repercussions on the quality of life of affected individuals but also directly affecting the lives of their relatives and caregivers [7], with an important economic burden involved [8].

TBI is triggered by a sudden event that elicits morphophysiological disturbances in the brain parenchyma, with variable impact depending on the location and extent of the injury. The deleterious events underlying TBI can be classified into two sequential stages: primary and secondary [9,10]. The primary injury is a disturbing event that occurs at the time of the initial trauma, causing an irreversible loss of tissue in the core of the lesion. The nature of the insult is highly relevant for a proper characterization of the levels of damage, early diagnosis, and therapeutic interventions. 

Depending on whether or not the skull is ruptured, the primary injury can be classified as a penetrating (open-head) or nonpenetrating (closed-head or blunt) lesion [9,10]. Penetrating injury is mainly defined by an open wound in the head caused by a foreign body, resulting in a focal disturbance that occurs along the path taken by the object through the tissue. It is associated with perforation or fracture of the skull, laceration of the meninges, and structural damage to nervous tissue [10]. Conversely, nonpenetrating injury is characterized by tissue damage caused by indirect impact without penetration of a foreign body into the brain. The skull may or may not be injured, but the meninges are not structurally disrupted [10]. 

Concerning the nature of trauma, nonpenetrating lesions can be classified into acceleration and non-acceleration injuries. While the first is associated with whiplash-type injury, resulting in the impact of the brain with the skull due to abrupt incidental acceleration or deceleration of the head, causing a contusion at the site of impact, as seen in blast injury [11], the latter is elicited by repeated blows to the head [12], resulting in deformation of the skull and causing focal localized damage to both meninges and brain tissue [10]. Mechanical tissue deformation, disturbance in the blood flow, osmotic/electrolyte imbalance, necrotic cell death, and the influx of inflammatory cells (neutrophils and monocytes) from bloodstream are hallmarks of the primary lesion in both animal models and humans, which is irreversible and amenable only to preventive measures to reduce the extent of damage [13].

The harmful effects following primary injury are not restricted to the site of the lesion. A primary lesion elicits a cascade of pathophysiological events that affects remote brain regions initially not affected, resulting in the so-called secondary injury, referred to as the additional damage that occurs after the primary insult following TBI. While the primary injury is the initial physical impact or event, the secondary injury involves a cascade of complex pathophysiological processes that can exacerbate the initial damage and lead to further neurological dysfunction and tissue loss [13,14].

Secondary brain injury can result from various mechanisms, including ischemia, excitotoxicity, oxidative stress, inflammation, and mitochondrial dysfunction in both animal models and humans [13,14]. These processes can cause a myriad of harmful events, such as brain edema, blood–brain barrier (BBB) disruption, increased intracranial pressure, metabolic disfunction, excitotoxicity, oxidative and cellular apoptosis or necrosis, ultimately leading to neurological disfunctions [13,15,16,17].

The secondary injury cascade typically unfolds over time, evolving from minutes to days after the initial insult. It can be influenced by factors such as systemic hypotension, hypoxemia, increased intracranial pressure, and metabolic imbalances [18]. The severity of the secondary injury and its impact on the patient’s outcome depend on various factors, including the nature and extent of the primary injury as well as the effectiveness of medical interventions to mitigate its progression [14].

Among the factors that contribute to secondary injury, brain edema, inflammatory response, excitotoxicity, and oxidative stress play a pivotal role [19,20,21,22], constituting an important field of investigation in both human subjects and animal models (Table 1 shows examples of studies evaluating animal models explored in the present study). In this context, our goal in the present work is to provide an updated review focusing some aspects related to TBI, with emphasis in the above-cited factors.

### 1.1. Brain Edema

One of the critical events following TBI is the formation of brain edema [37], which is characterized by an increased water content in the intracellular and/or extracellular spaces of the brain, resulting in swelling of the organ [38]. Classically, brain edema is classified into either vasogenic or cytotoxic, both being elicited hours after TBI [39].

Vasogenic edema is a type of cerebral edema characterized by the accumulation of extravascular fluid in the extracellular space due to increased permeability of the BBB caused by damage to cerebral blood vessels, resulting in plasma extravasation and edema formation [40]. Vasogenic edema is a common complication in various brain conditions such as traumatic brain injury, acute ischemic stroke, brain tumors, and infections [41].

The pathogenesis of vasogenic edema involves a series of complex events. Injury to cerebral blood vessels triggers an inflammatory response, leading to the release of chemical mediators such as cytokines, interleukins, and growth factors [42]. These mediators increase vascular permeability and facilitate fluid extravasation into the surrounding brain tissues.

The main consequence of vasogenic edema is increased intracranial pressure, which can cause severe neurological symptoms and even life-threatening conditions [43]. Common symptoms include severe headache, altered mental status, focal neurological deficits, seizures, and, in severe cases, coma.

Diagnosis of vasogenic edema is based on clinical evaluation of symptoms and imaging studies such as computed tomography (CT) and magnetic resonance imaging (MRI) [44]. These imaging techniques can identify the presence of cerebral edema and help determine the underlying cause. The treatment of vasogenic edema aims to reduce intracranial pressure and control symptoms. This often involves the use of diuretic medications such as mannitol, which help reduce fluid volume in the brain [45]. In addition, decompressive craniectomy and osmotherapy are also adopted as treatment for cerebral edema [46].

Cytotoxic edema, conversely, is a type of cerebral edema characterized by the accumulation of fluid within brain cells due to alterations in cell membrane function [43]. Unlike vasogenic edema, which involves fluid extravasation from blood vessels, cytotoxic edema results from cellular swelling and increased intracellular fluid content [39].

The main cause of cytotoxic edema is the disruption of ion homeostasis, particularly the failure of the Na^+^/K^+^ pump and subsequent intracellular sodium (Na^+^) and water accumulation [39], leading to cellular swelling, impaired cellular function, and compromised energy metabolism [47]. In addition, oxidative stress and mitochondrial dysfunction contribute to the development and progression of cytotoxic edema [48].

In the case of edema formation after a TBI, the membrane protein aquaporin 4 (AQP4), responsible for cellular water transport, shows changes in its expression depending on the type of edema to be formed, as observed in rats in a model of TBI due to cranial perforation. While in vasogenic edema there was downregulation, in cytotoxic edema, there was an upregulation of AQP4 [49]. In this sense, the silencing or attenuation of AQP4 gene expression appears to be effective in reducing the formation of edema after TBI [50,51,52].

Cytotoxic edema has significant implications for neuronal viability and brain function. The increased intracellular volume can result in cellular damage, impaired neurotransmission, and disrupted neuronal signaling pathways [43]. If left untreated, cytotoxic edema can lead to irreversible cellular injury and neurologic deficits.

The diagnosis of cytotoxic edema relies on advanced imaging techniques such as MRI and diffusion-weighted imaging (DWI), which can demonstrate cellular swelling and restricted diffusion within affected brain regions [44,53]. Clinical evaluation and identification of the underlying cause are crucial for accurate diagnosis.

The treatment of cytotoxic edema aims to address the underlying condition and mitigate cellular swelling. Management strategies may include optimizing cerebral perfusion, reducing intracellular Na^+^ levels, and promoting cellular recovery [38]. Additionally, targeted therapies using conivaptan (a vasopressin V1a receptor antagonist) as well as the regulation of aquaporin molecules show promise in attenuating cytotoxic edema [30,54,55].

### 1.2. Types and Localization of Traumatic Lesions following Head Injury

Post-traumatic brain edema impacts severely the nervous tissue, with injuries following TBI being classified into two main types: hemorrhagic and non-hemorrhagic. The first one occurs when blood vessels in the brain or the space between the brain and its membranes rupture, resulting in bleeding inside the skull, which can lead to increased intracranial pressure [56]. The second one, conversely, does not involve bleeding in the brain, being characterized by damage to brain tissues without blood vessel rupture. This type of injury may include brain contusions, concussions, and diffuse axonal injuries [57].

Hemorrhagic injuries following TBI are characterized into four types: epidural or subdural hematoma and intracerebral or subarachnoid hemorrhage, leading to high rates of morbidity and mortality globally, especially in adults [58]. Epidural hematoma is characterized as a collection of blood between the inner surface of the skull and the dura mater. It is usually caused by a tear in the middle meningeal artery, often resulting from a skull fracture. Epidural hematomas can rapidly lead to increased intracranial pressure, requiring urgent surgical intervention [59]. Subdural hematoma occurs when blood collects between the dura mater and the brain’s surface, which is often caused by tears in the veins that bridge the brain and the dura. Subdural hematomas can be acute (developing rapidly) or chronic (developing slowly over time) and may require surgical drainage in severe cases [59]. Intracerebral hemorrhage, in turn, involves bleeding directly within the brain tissue itself, which is often caused by the rupture of small blood vessels due to the force of impact during a head injury. The location and size of the intracerebral hemorrhage determine its severity and potential impact on brain function, whilst subarachnoid hemorrhage involves bleeding into the space between the arachnoid membrane and the pia mater, being typically caused by the rupture of an intracranial aneurysm or head trauma. Subarachnoid hemorrhages can lead to a potentially dangerous increase in intracranial pressure and may require urgent medical attention [60].

Concerning non-hemorrhagic lesions, there is no bleeding within the brain tissue, which can result from a blow to the head, a fall, or any other impact that causes the brain to move inside the skull, resulting from other types of damage caused by trauma. Common non-hemorrhagic lesions are concussion, contusion and diffuse axonal injury. The first one is the mildest form of traumatic brain injury, which is often caused by a direct blow to the head, resulting in a temporary disruption of brain function; its main symptoms include headache, nausea, vomiting and dizziness [61]. The second one, in turn, is characterized by a bruise on the brain caused by the brain impacting the inner surface of the skull during trauma. Contusions can occur at the site of impact (coup injury) or on the opposite side (contrecoup injury), and they may lead to localized brain tissue damage [62]. Diffuse axonal injury occurs when there is widespread damage to the brain’s axons due to strong rotational forces during the injury, affecting multiple areas of the brain and resulting in widespread functional impairment [63].

Understanding the type of brain lesion is crucial in determining the appropriate medical management and treatment plan for a traumatic brain injury. Imaging techniques like CT scans and MRI are used to diagnose and evaluate the extent of these lesions, helping healthcare professionals make informed decisions regarding patient care and prognosis [64,65].

Another factor that is important regarding outcomes following moderate and severe TBI is related to the localization of the lesion, which is correlated with the prediction of the prognosis after head injury [66]. Midline and brainstem lesions pose more severe threats due to their impact on vital functions and higher mortality rates. 

Lesions located in the midline or brainstem are particularly concerning due to their critical role in essential physiological functions, since these structures govern vital functions, such as breathing, heart rate, consciousness, and other autonomic functions [67]. The impairment of vital functions related to midline and brainstem lesions can disrupt vital autonomic functions, leading to altered consciousness, respiratory disturbances, and cardiovascular instability. Such impairments may increase the risk of complications and worsen the overall prognosis [68]. In addition, the proximity of these lesions to crucial life-sustaining centers increases the likelihood of life-threatening consequences, making these injuries more fatal compared to other brain injury types.

Cortical and subcortical lesions, in turn, refer to injuries occurring in various regions of the cerebral hemispheres, including the frontal, temporal, parietal, and occipital lobes. Lesions in these areas can lead to a wide range of neurological deficits, including impaired memory, attention, language, and motor function, with the severity of cognitive and motor impairments varying based on the specific location and extent of the lesion [69,70]. However, unlike midline and brainstem injuries, some cortical and subcortical lesions may have a higher potential for recovery and rehabilitation [71].

It is worth noting that every head injury is unique, and prognostic outcomes can vary significantly from case to case. The management of head injuries typically involves prompt and appropriate medical care, which may include imaging studies (CT scans, MRI) to assess the extent of the damage, surgery if necessary, and ongoing monitoring and rehabilitation [65]. Multidisciplinary approaches, including neurosurgery, neurology, and rehabilitation specialists, are essential to optimize outcomes and improve the quality of life for individuals affected by traumatic brain injuries.

### 1.3. Inflammation

Inflammation is defined as a cardinal defense mechanism consisting of a series of physiological humoral and cellular responses induced by disturbances in the integrity of tissue homeostasis mediated by pathogens, physical agents (burn, radiation, traumatic lesions), toxins, vascular alterations, tissue necrosis and/or immunological reactions [72,73,74], aiming to safeguard the tissue and promoting healing [75,76]. Such characteristics are associated with vascular changes that occur during the inflammatory process, with inflammation being classified as acute or chronic, according to its duration [77].

Inflammatory response involves the participation of humoral (cytokines, growth factors), cellular (lymphocytes, macrophages, vascular endothelial cells, fibroblasts) and extracellular matrix (collagen, elastin, fibronectin) components [78]. The interchanged actions of these factors have the purpose of safeguarding the organism from the harmful actions of detrimental elements and, at the same time, reconstituting tissue integrity through repair and regeneration mechanisms [78].

Acute inflammation emerges shortly after the injury; it is mainly characterized by the infiltration of leukocytes and vasodilatation, whilst chronic inflammation arises later from a more specific immune response [79]. However, in some pathological conditions, an exacerbated inflammatory response, whether acute or chronic, can increase tissue damage. In this scenario, a proper understanding of the basic mechanisms of the inflammatory response is critical for a better characterization of the pathophysiology specifically associated with TBI.

Several inflammatory mediators, such as chemokines, proteases, cytokines, and reactive oxygen species (ROS), are synthetized following TBI, contributing to the spreading of the lesion, resulting in secondary cell damage [80,81]. In the nervous system, these substances are synthesized and released during the inflammatory process by astrocytes and microglia, which significantly contribute to the expansion of the injury observed after the primary trauma in animal models, generating a process of wound amplification that is more damaging to pathological progression than the primary injury itself [31,82,83,84].

Astrocytes, a component of macroglia, are directly involved in the maintenance of the homeostasis of the nervous tissue by regulating ionic and hydric levels as well as contributing to the structural maintenance of the BBB through the interaction between the astrocytic end feet and endothelial cells [85,86]. In addition, these cells play a pivotal role in the uptake of glutamate and the regulation of calcium (Ca^2+^) intracellular signalizing [87]. Following TBI, astrocytes are quickly activated, undergoing significant structural changes, with shortening of its processes and swelling of the cell body, assuming a hypertrophic shape. In their activate state, astrocytes act phagocyting debris and releasing cytokines, chemokines and inflammatory mediators such as tumor necrosis factor-alpha (TNF-α), cyclooxygenase-2 (COOX-2) and matrix metalloproteinase 9 (MMP-9) in order to sustain the inflammatory process [88,89]. In addition, astrocyte proliferation around the site of the primary lesion forms the so-called glial scar, isolating the wounded tissue and safeguarding the normal tissue around the injured region following both traumatic lesion [90] and also in response to the implant of artificial devices [91]. Although beneficial to the tissue, the glial scar creates a mechanical barrier that interferes with the process of axonal regeneration, with implications for the functionality of the region [92].

Microglia, a class of resident cells of the nervous system, plays a critical role during inflammatory response by releasing pro-inflammatory mediators following noxious insults [15]. Microglial cells are quite sensible to a minimal disturbance in extracellular milieu, responding quickly and vigorously to the altered state [93]. Such a process is accompanied by morphological changes, with microglia assuming an amoeboid morphology [94]. During this event, chemical mediators released by these cells, such as prostaglandins, cytokines and chemokines, further increase the inflammatory process. The microglial activation underlying a TBI results in the amplification of the inflammatory response, since it induces the synthesis and release of TNF-α and interleukins such as IL-1β and IL-6 [95].

**Figure 1:**
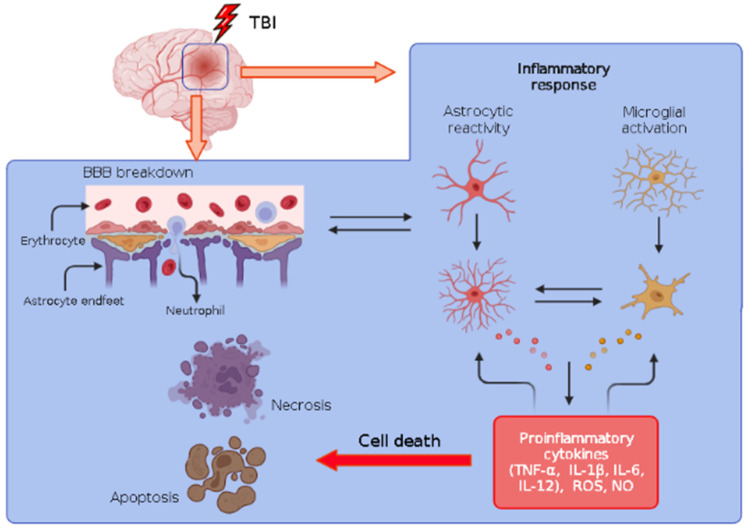
Summary of the general events associated with inflammatory response following a TBI. Traumatic lesion results in blood–brain barrier (BBB) breakdown, causing influx of neutrophils into the nervous tissue. In a few minutes, both astrocytic reactivity and microglial activation induce the release of pro-inflammatory cytokines such as tumor necrosis factor-alpha (TNF-α), interleukins (IL-1β, IL-6, IL-12), reactive oxygen species (ROS) and nitric oxide (NO), which further induce the release of these substances by the cells, establishing an inflammatory feedback loop, ultimately leading to tissue impairment and both necrotic and apoptotic death. Figure created in BioRender.com (accessed on 7 June 2023).

TNF-α, in particular, has an important role during the acute inflammatory process, since it induces the expression of IL-1 and IL-6; IL-1, in turn, induces both TNF-α and IL-6. Accordingly, after a TBI, an initial upregulation of cytokines leads to the attraction of other inflammatory mediators to the core of lesioned tissue, triggering an inflammatory loop [96]. Whether the inflammatory process becomes persistent, lasting for weeks or even months, a chronic inflammation is established. In that condition, cytokine interactions result in monocyte migration to the site of the lesion, where cytokines such as interferon-γ (IFN-γ) and monocyte chemoattractant protein-1 (MCP-1) further activate the macrophages, which accumulate in the inflammatory site. The macrophages, in turn, contribute to the exacerbation of the inflammatory process by releasing chronically TNF-α and IL-1. In addition, interleukins such as IL-2, IL-4 and IL-7 also contribute to the increase of the inflammation [97]. Activated microglia also induces the production of nitric oxide (NO) and ROS, which impair both metabolism and cell structure, resulting in apoptosis and tissue failure [98]. Figure 1 summarizes the general events associated with the inflammatory response following a TBI.

### 1.4. Excitotoxicity

Excitotoxicity, a phenomenon associated to damage caused by an excessive concentration of glutamate and its agonists in the nervous parenchyma, which leads to an increase in the influx of Ca^2+^ to the cell and its impairment due to the high intracellular overload of this ion [99], is recognized as a critical factor in triggering a plethora of events following TBI [100]. Its mechanism involves the stimulation of a myriad of deleterious biochemical cascades, which are responsible for cellular degeneration through the activation of several enzymes such as proteases, lipases, phosphatases and endonucleases, triggering the process of generating free radicals that affect both the structure and cell physiology, ultimately resulting in cell impairment [101,102,103].

Imbalanced concentrations of glutamate extracellular following TBI induce the activation of Na^+^ and Ca^2+^ channels in the cell membrane [101], resulting in a rapid influx of these ions and causing an increased release of glutamate by the cell, which produces neurotoxicity by overstimulating NMDA receptors, leading to more Ca^2+^ influx, establishing a detrimental looping that ultimately results in excitotoxic cell death [13]. Moreover, the excessive influx of Ca^2+^ following TBI contributes to mitochondrial failure with an overgeneration of ROS, leading to the breakdown of the cell membrane [13].

Apoptosis is one of the key events triggered by excitotoxicity through the activation of caspases, which is an evolutionary conserved family of aspartic acid-specific cysteine-dependent proteases directly involved in the apoptotic process and inflammatory responses initiated by the increase in intracellular Ca^2+^ levels [102]. Functionally, caspases are classified into two types: (i) initiators (caspases 2, 8, 9 and 10), expressed in healthy cells as inactive zymogens, which activate effector caspases by cleaving specific sites, and (ii) effectors (caspases 3, 6 and 7), which act cleaving other protein substrates, resulting in cell death [104]. The initiation process of the apoptotic cascade occurs from a specific stimulus that activates the initiator caspases, which in turn activate the effector caspases, resulting in a cascade of events that compromises the cellular structural and metabolic mechanisms, ultimately causing its death [104].

One of the most important caspases in the process of apoptotic death is caspase 3. Its activation triggers the proteolysis of DNA repair proteins and degradation of cytoskeletal proteins such as spectrin, causing structural changes that result in apoptosis [23,105]. TBI induces mitochondrial disruption, which is a critical event associated with apoptotic cell death [106]. Initially, mechanical perturbations induce Ca^2+^ overload and opening of the mitochondrial membrane permeability transition pore (MPTP) [107], resulting in mitochondrial swelling and the consequent impairment of its transmembrane potential [23]. Next, cytochrome c (cyto c) located on the inner mitochondrial membrane is released into the cytoplasm, coupling to the caspase activating factor Apaf-1 and initiator caspase 9, resulting in the induction of pro-caspase 3. After activation, caspase 3 contributes to the impairment of proteins and enzymes related to the maintenance of structural integrity, signal transduction, transcription and also DNA repair [108]. Other caspases are also involved in the pathophysiology of TBI, indicating that several pathways are activated in the process [109].

Another important protease, calpain, directly regulated by adequate levels of Ca^2+^, is induced after the activation of glutamatergic receptors [110]. It plays an important role in the degradation of several structural proteins in the neuron, such as tubulin, tau, and microtubule-associated protein (MAP), cleaving and inactivating the Na^+^/Ca^2+^ exchanger of the plasma membrane in neurons, which is a fundamental membrane protein for maintaining intracellular Ca^2+^ homeostasis, causing overload of this ion and resulting in necrotic and apoptotic cell death [111].

Phospholipases are a group of enzymes involved with normal physiological aspects such as metabolism, the production of bioactive lipid mediators and host defense [112]. In a pathological condition induced by TBI, however, their overexpression is involved with cell membrane breakdown, inflammatory response, and oxidative stress [113,114]. For instance, traumatic spinal cord injury induces the activation of phospholipase A2 (PLA2) through injury mediators such as inflammatory cytokines, ROS and excitatory amino acids. Such an event can induce the overactivation of PLA2, further stimulating ROS synthesis and membrane phospholipids impairment, ultimately causing cell dysfunction and death [113].

**Figure 2:**
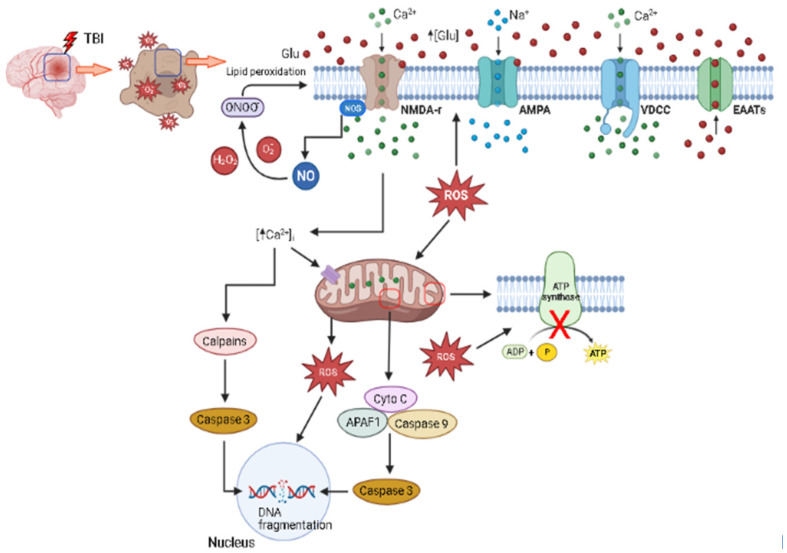
Summary of the general events associated with excitotoxicity following a TBI. Traumatic injury induces massive glutamate excitotoxicity that activates NMDA and AMPA receptors, inducing an excessive influx of Ca^2+^ that accumulates in the mitochondria, which responds by producing ROS. In addition, Ca^2+^ induces an impairment of ATP synthesis and release of cyto c into cytoplasm, activating the APAF1/caspase 9 complex, inducing caspase 3, which induce apoptosis by DNA fragmentation. Ca^2+^ also activates the nitric oxide synthase (NOS) enzyme, leading to NO synthesis, inducing the production of oxygen-derivate species that attack the cell membrane. Excessive Ca^2+^ overload activates calpains, and the intracellular increase in ROS production causes damage to DNA, lipids, and proteins, ultimately impairing cell function. Figure created in BioRender.com (accessed on 7 June 2023).

By the same token, TBI induces the increased expression of bradykinin B2 receptor, which regulates the PLA2 Ca^2+^-mediated signaling pathway, generating PLA2 upregulation [114], which amplifies inflammation and results in cell degeneration [115]. The blockade of both PLA2 and bradykinin B2 receptors results in a protective effect in nervous tissue following TBI by decreasing edema and improving behavioral outcomes in a rat model, pointing to a detrimental effect of acute inflammation induced by traumatic injury [114]. Figure 2 summarizes the cell disturbances induced by excitotoxicity.

### 1.5. Oxidative Stress

Oxidative stress is a biochemical event that naturally occurs in organisms. Classically, it is defined as an imbalance between the antioxidant competence and reactive oxygen/nitrogen species (RONS) [116]. The former consists of enzymatic and non-enzymatic players, such as uric acid, glutathione, ascorbic acid, catalase, glutathione peroxidase (GPx), superoxide dismutase (SOD), and several others [117,118]. The latter, in turn, involves the production of free radicals and non-radical species exemplified below. Such prooxidant agents may cause damages to the cellular macromolecules, generating DNA adducts, and oxidize lipids present on the cell and organelles membrane, called lipid peroxidation, and also protein carbonylation [119,120]. In contrast to these classical mechanisms and definition, the endoplasmic reticulum stress has been inserted on oxidative stress events that can lead to protein compromise, being characterized by the production and release of misfolded proteins in the cell [121].

In addition to that, it is important to state that there is a dual understanding of oxidative stress in the literature. In one hand, some researchers only consider an effective oxidative stress state when the before-mentioned imbalance results in the significant increase in end-products, such as malondialdehyde in lipid peroxidation, carbonylated proteins and DNA adducts. On the other hand, others researchers consider the simple imbalance between both biochemical players as features of such a state (see [122] for a review). In any scenario, the discussion of this mechanism involved in TBI considering both understandings will be addressed, highlighting the biochemical players evidenced in the literature.

Severe disturbances in the central nervous system (CNS), such as a severe TBI, cause significant changes in cellular redox homeostasis [5]. Among the major contributors to the pathological condition following TBI oxidative stress, the pivotal ones involve the production of derivatives of ROS (oxygen-free radicals, peroxynitrite, superoxide, hydrogen peroxide, and NO) during the pathological insult [123,124], which can induce the degeneration of the structural and functional integrity of cells, and modification of proteins, nucleic acids, and lipids [125], ultimately leading to both necrotic and apoptotic cell death.

Physiologically, cells have a myriad of antioxidant mechanisms against the deleterious action of noxious elements [126]. Nonetheless, in some pathological conditions such neurodegenerative diseases, stroke, and TBI, the mechanisms of cell redox balance are unable to maintain such substances under physiological levels, resulting in oxidative stress, with the production of oxidizing ROS suppressing the body’s defenses for an unbalance between the production of antioxidants agents and free radicals [127,128]. In such circumstances, depletion of the endogenous antioxidant system results, as decreased levels of catalase, GPX, and SOD enzymes lead to excessive ROS generation that can result in neural dysfunction and death [128,129]. This process is also responsible for protein oxidation, the peroxidation of cell structures and DNA damage, leading to a loss of mitochondrial function, inducing Ca^2+^ overload and resulting in ROS synthesis that amplifies the metabolic failure [129,130].

The excessive production of free radicals is one of the main elements triggering neurotoxicity [131]. Such substances are very important for the damaging mechanisms during glutamate-mediated excitotoxic injury following TBI [123,125]. The resultant effect of ROS production after a TBI is associated with increased damage to the brain parenchyma with neuronal degeneration, increased inflammatory response, and loss of physiological functions [131].

Glutamate release and microglial activation induce neuroinflammation by an excessive liberation of NO mediators, facilitating neuronal death; nitric oxide synthase (NOS) enzyme, in turn, can induce lipid peroxidation and further promote glutamate release, establishing a deleterious feedback loop [129,130]. Compelling evidence show that the activation of NMDA receptors plays a key function in the synthesis of free radicals in at least three pathways: (i) activation by Ca^2+^ of PLA2 with release of arachidonic acid and formation of free radicals; (ii) conversion of xanthine dehydrogenase enzyme to xanthine oxidase by influx of Ca^2+^ with production of ROS [132]; and (iii) synthesis of NO by the activation of the NOS, which is induced by influx of Ca^2+^ through the stimulation of NMDA receptors [133]. It is believed that NO can react with the superoxide anion to produce the biological oxidant peroxynitrite [134]; this process can lead to the formation of potent free radicals such as the hydroxyl radical and the production of NO, which plays an important role in the neurotoxicity mechanisms of glutamate. The toxic effects of NO are mainly mediated by its oxidation products [135]. In addition, arachidonic acid metabolism may be an essential source of free radicals as well [136].

Overall, ROS are spontaneously produced by mitochondria in healthy individuals. During the electron transport chain’s normal activity, instead of reducing oxygen to water, superoxide anion (O_2_^−^) is formed [137]. Notwithstanding, after a TBI, mitochondria significantly increase ROS production, causing acute oxidative stress [138]. Cells are extremely dependent on the energy produced in mitochondria to maintain their physiological functions, and mitochondrial dysfunction tends to be catastrophic [139]. During a TBI, this organelle is one of the cellular structures more susceptible to disturbance, which is induced by Ca^2+^ influx overload triggering alterations in the transport electron chain and resulting in bioenergetic failure, being involved with apoptotic cell death due to the releasing of cyto c into cytoplasm [23]. Under massive Ca^2+^ loads, opening of the MPTP results in the release of mitochondrial Ca^2+^ and other molecules. Such a phenomenon discharges and uncouples the electron transport chain, which can cause cell necrosis or apoptosis [140].

Mitochondrial DNA (mtDNA) is especially affected in cases of oxidative stress in addition to being close to where ROS are produced. Unlike nuclear DNA, mtDNA does not have histones and therefore is more susceptible to ROS attacks [138]; a continuous exposure of mtDNA to ROS causes damage and consequent mutations, which in turn lead to respiratory chain dysfunction, making mitochondria less efficient at energy production and increasing ROS production [141]. The acute exposure of mitochondria to ROS can trigger significant morphological changes such as a decrease in cristae and its aggregation. Modifications of this nature can impair the mitochondrial ability to produce adenosine triphosphate (ATP) [142].

ROS are not only synthesized in mitochondria; there is also a family of enzymes called NADPH-oxidases (NOX) located in the cell membrane that also produce these molecules, and they have a key role in altered states of the brain [143]. The NOX enzyme family can be divided into NOX1, NOX2, NOX3, NOX5 and DUOX1/DUOX2, which produce O_2_**^−^**, and NOX4, which produces hydrogen peroxide (H_2_O_2_). Nevertheless, NOX2 and NOX4 are the isoforms that seem to play a critical role in TBI cases [143]. Li et al. [144] observed that after a TBI, there is a significant increase in the expression of NOX2 with a peak between 12 and 24 h after the injury, while NOX4 has higher levels between 24 and 48 h after the trauma. Therefore, transient increases in NOX expression in the brain after TBI correlate with oxidative stress, protein, cell membrane and DNA damage, inflammasome activation and microglial activation [145].

The pharmacological inhibition of NOX by apocynin 15 min before TBI provoked an attenuation of ROS production in the hippocampus of rats and consequently reduced neuronal death and microglia activation. In this way, NOX inhibition may have a therapeutic role in reducing the damage linked to TBI [24]. In this context, some natural substances have been studied aiming at neuroprotection in cases of oxidative stress caused by TBI. For instance, puerarin [146], astragaloside IV [147], N-acetylcysteine amide [148], and resveratrol [149] can act by different mechanisms, reducing the deleterious effects after a TBI. Importantly, anti-inflammatory, antioxidant and anti-apoptotic agents are crucial for the bioavailability of therapeutics after a TBI [150]. In addition, aerobic physical activity also can be employed to attenuate oxidative stress by controlling ROS levels through an upregulation of endogenous antioxidant defenses, especially GPx levels [151,152].

**Figure 3:**
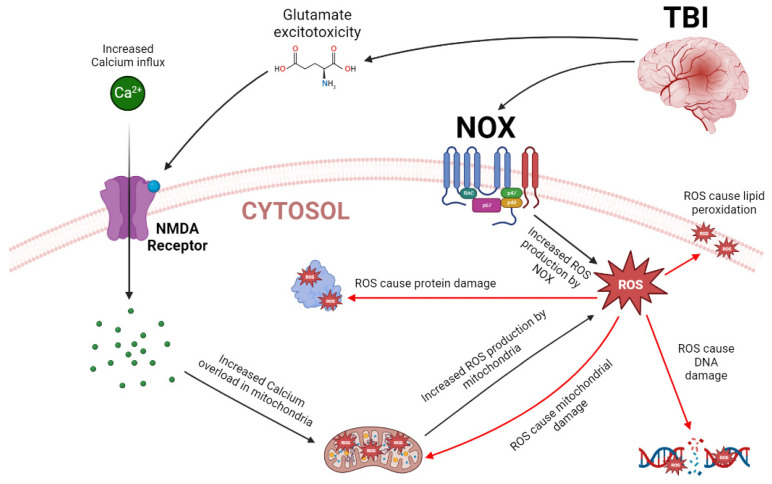
Summary of the general mechanism associated with oxidative stress following a TBI. Traumatic injury induces massive glutamate excitotoxicity that causes an excessive influx of Ca^2+^ that accumulates in the mitochondria, which responds by producing ROS. In parallel, a TBI causes an increased expression of NOX, which also produce ROS. The intracellular increase in ROS production causes damage to DNA, lipids, and proteins, ultimately impairing cell function. Figure created in BioRender.com (accessed on 7 June 2023).

The general mechanisms of oxidative stress following a TBI are summarized in the Figure 3.

### 1.6. Metabolic Disturbances

TBI can result in significant metabolic disturbances due to changes in brain function and the physiological stress associated with the injury. Such disturbances can have a significant impact on patient recovery and prognosis. Some of the key metabolic disturbances observed following TBI include glucose metabolism dysfunction, electrolyte imbalances, increase in lipid peroxidation, endocrine dysfunction, and alterations in neurotransmitter metabolism [153,154].

One of the most common metabolic disturbances following TBI is glucose metabolism dysfunction. The brain primarily relies on glucose as its energy source, and TBI can disrupt this energy supply. The injury can lead to an increased metabolic demand of the brain, resulting in hypoglycemia or decreased brain glucose levels, with cognitive decline associated [155]. Furthermore, reduced insulin sensitivity was observed following TBI, indicating that insulin resistance may occur, further compromising glucose metabolism [156]. These metabolic alterations can lead to cerebral dysfunction, cognitive impairment, and worsened clinical outcomes [153].

In addition to glucose metabolism dysfunction, electrolyte imbalances are reported after TBI [157]. Brain injury can cause alterations in Na^+^, K^+^, Ca^2+^, and other electrolyte levels, which can be used as a prognostic factor for mortality and morbidity following TBI [158,159,160]; for instance, an excessive release of antidiuretic hormone (ADH) may cause the syndrome of inappropriate antidiuretic hormone secretion (SIADH), leading to water retention and dilutional hyponatremia. Conversely, an excessive release of adrenocorticotropic hormone (ACTH) may result in cerebral salt-wasting syndrome (CSWS), causing excessive urinary Na^+^ loss. In addition, patients diagnosed with hypokalemia following TBI present an increased risk of morbidity and mortality resulting from alterations in fluid balance [160,161]. So, it is essential to monitor and correct any electrolyte imbalances to promote adequate neurological protection.

Furthermore, TBI can also lead to alterations in neurotransmitter metabolism by affecting the synthesis, release, reuptake, and metabolism of neurotransmitters such as serotonin, dopamine, GABA and glutamate [162,163,164]. These alterations can impact mood, behavior, cognitive function, and may even contribute to the development of neuropsychiatric disorders following the injury [165].

The careful monitoring of metabolic parameters is crucial in the management of TBI patients. This involves monitoring glucose levels, electrolytes, and other relevant metabolic parameters. Depending on the severity and individual needs of the patient, interventions such as glucose administration, correction of electrolyte imbalances, and nutrition therapy may be adopted [166]. The proper monitoring and treatment of these metabolic disturbances is essential to optimize recovery and prognosis in TBI patients.

### 1.7. Signaling Pathways

A complex cascade of molecular and cellular events contributes to the neuropathology and neurological dysfunction observed following TBI. These pathways play critical roles in cell survival, inflammation, oxidative stress, neuronal plasticity, and cognitive function.

One important signaling pathway implicated in TBI is the nuclear factor-kappa B (NF-κB) pathway [167]. NF-κB is a transcription factor that regulates the expression of genes involved in inflammation and cell survival, acting as a downstream element for the stimulation of several receptors, such as tumor necrosis factor receptor-associated factor 6 (TRAF-6) and Toll-like receptor 4 (TLR-4) in humans and animals that suffered TBI, with its inhibition reducing apoptotic cell death and levels of inflammation after injury. The activation of NF-kB in neuronal and glial cells is associated with neuroprotective activity and neurodegenerative diseases, including TBI [168].

Another crucial pathway in TBI is the mitogen-activated protein kinase (MAPK) pathway. The MAPK pathway consists of several protein kinases, including extracellular signal-regulated kinase (ERK), c-Jun N-terminal kinase (JNK), and p38 MAPK. These kinases play key roles in cellular responses to stress, inflammation, and apoptosis. Activation of the MAPK pathway following TBI has been associated with neuronal apoptosis, neuroinflammation, and cognitive impairments [82,167].

Furthermore, the phosphoinositide 3-kinase (PI3K)/Akt pathway has been implicated in TBI pathology. This pathway regulates cell survival, neurogenesis, and synaptic plasticity. Activation of the PI3K/Akt pathway has been shown to promote neuronal survival and enhance recovery after TBI, whilst its inhibition can exacerbate neurodegeneration and functional deficits [167]. In this regard, the activation of Brain-Derived Neurotrophic Factor (BDNF)/receptor tyrosine kinase (TrkB) signaling via PI3K/Akt/MAPK appears as a promising neuroprotective alternative for the treatment of TBI [169].

BDNF has been the subject of several studies aimed at neuroprotection after a TBI incident. This molecule binds to two types of receptors: the TrkB, which has the highest affinity, and also the pan-neurotrophin receptor p75NTR, a member of the tumor necrosis factor receptor (TNFR) family, which has the lowest affinity [170]. BDNF/TrkB activation stimulates MAPK, phospholipase C-γ (PLC-γ), and PI3K/mTOR pathways. These pathways together lead to the expression of genes related to important roles in brain functioning, such as neuronal survival, dendritic growth, axonal sprouting, and synaptogenesis [171]. BDNF has its levels increased in cerebral fluid between 1 and 6 h after a brain trauma in rats [172]. Increased BDNF levels of cerebral fluid after an episode of TBI may be detrimental due to injury and the subsequent increase in pro-apoptotic BDNF target receptors [173].

Another signaling pathway that has drawn the attention of scientists working in the field of TBI is the mechanistic target of rapamycin (mTOR) pathway. In non-pathological conditions, this pathway plays a crucial role in development, synaptic plasticity, memory and metabolic regulation of the CNS [174]. However, abnormalities of the mTOR signaling pathway may contribute to the development of a plethora of CNS diseases, such as neurodegenerative and neuropsychiatric disorders [175].

It was described by Chen et al. [176] that between 30 min and 24 h after a TBI in rats, there was an increase in the levels of phosphorylated mTOR (active form) and also of its downstream target, p70S6K, in the ipsilateral damaged hippocampus and parietal cortex. Considering that the activation of the mTOR pathway promotes cell and synaptic growth and repair, it can be assumed that the activation of these pathways can act in the remodeling of neuronal circuits and synaptic plasticity after TBI. Alternatively, activation of this pathway can also induce aberrant budding, leading to the post-traumatic seizures that are often seen after TBI [177].

Interestingly, the pharmacological inhibition of the mTOR pathway in animal models of TBI is beneficial for ameliorating TBI-associated symptoms and inflammatory responses [178]. The inhibition of mTOR by KU0063794 caused a reduction in TBI-related inflammatory parameters: 24 h after lesion, mice that had mTOR inhibition had significantly lower levels of TNF-α and IL-1β when compared to mice in the control group [179]. Similarly, mice that had the mTOR pathway inhibited by rapamycin had lower levels of astrogliosis in the hippocampus and better performance on behavioral tests of learning [180].

The Janus kinase/signal transducer and activator of transcription (JAK/STAT) is one of the main pathways activated by cytokine receptors and growth factor receptors, and it is responsible for the signal transduction from the cellular surface to the nucleus [181]. It has been reported that 3 h after TBI induction by cortical compression in rats, there is increased activation of the JAK2/STAT pathway in neurons and astrocytes, which is related to the increase in the production of inflammatory cytokines, secreted by T-cells and macrophages, after experimental brain injury [182].

From the same perspective, Oliva et al. [183] describe that Western blot analyses in the hippocampus of rats indicated that STAT3 phosphorylation increased significantly in 30 min and lasted 24 h after TBI. A significant increase in the phosphorylation of cytokine receptor glycoprotein 130 (gp130) and Jak2 was also observed. It is important to stress that the hyperactivation of the JAK/STAT pathway after TBI can cause an abnormal increase in neuronal firing, which may be related to changes in the expression of GABA_A_R subunits. In this context, the inhibition of the JAK/STAT pathway by WP1066 proved to be effective in reversing the decrease in transcription of the α subunit of GABA_A_R caused by TBI and also in improving vestibular motor recovery [184].

In a severe stretch brain injury model in rats, which causes diffuse axonal injury, there was increased activation of the myelin growth inhibitor rtn4 (Nogo-A), targeting and upregulating GTPase RhoA (ras homologous gene family, member A), which is related to the process of neurodegeneration. Is important to say that the triggering of RhoA pathway activation is caused by the increase in pro-inflammatory cytokines that occurs after TBI, mainly IL-1 [185].

The nuclear factor erythroid 2-related factor 2 (Nrf2) pathway is a relevant defense mechanism that regulates several detoxifying, oxidative, and anti-inflammatory genes expression, and because of this, it has been identified as a potential therapeutic target to mitigate the secondary damage caused by TBI [186]. After TBI, the nuclear Nrf2 protein level was significantly increased, and the mRNA levels of both products of transcription activation HO-1 and NQO1 are also upregulated. This suggests that there is a correlation between TBI and increased activity in this pathway, which may be a defense mechanism against oxidative stress and inflammation caused after lesion [187].

Evidence supporting this hypothesis is pointed out by Bhowmick et al. [188], where mice that suffered knockout for the Nrf2 gene had exacerbated brain damage as shown by the increased oxidative stress markers, pro-inflammatory cytokines, and apoptosis markers at 24 h after TBI. These results show the importance of the Nrf2 pathway in attenuating the deleterious effects caused by the pathophysiology of TBI.

Understanding these signaling pathways provides insights into the molecular mechanisms underlying TBI pathology and opens avenues for potential therapeutic interventions. For instance, targeting the NF-κB pathway could help modulate the inflammatory response, while modulation of the MAPK pathway might alleviate neuronal apoptosis and cognitive impairments. Activation of the PI3K/Akt pathway could promote neuroprotection and enhance functional recovery after TBI.

**Figure 4:**
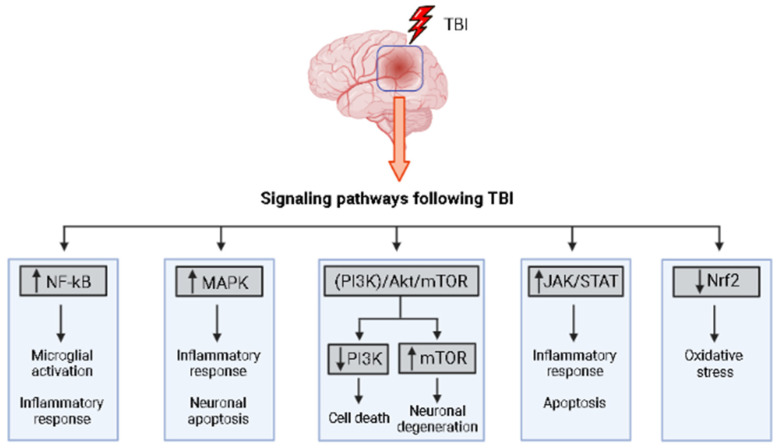
Summary of signaling pathways following a TBI. Nuclear factor-kappa B (NF-κB) signaling pathway, mitogen-activated protein kinase (MAPK) pathway, PI3K/Akt/mTOR signaling pathway, Janus kinase/signal transducer and activator of transcription (JAK/STAT) pathway, nuclear factor erythroid 2-related factor 2 (Nrf2) pathway. Figure created in BioRender.com (accessed on 27 July 2023).

Figure 4 summarizes the signaling pathways following a TBI.

## 2. Biomarkers following TBI

According to the Food and Drug Administration (FDA), biomarkers can be defined as a “characteristic that is measured as an indicator of normal biological processes, pathogenic processes, or responses to an exposure or intervention, including therapeutic interventions”. Recent advances in its characterization have been obtained in both humans and animal models [189,190,191,192].

Among the biomarkers reported in the literature in recent decades, there are neurodegeneration markers (tau and amyloid-beta 12), autophagy and cell destruction markers (Beclin-1 and LC3B), and inflammatory markers (GFAP, TNF-α, IL-6, NO) with special attention paid to the S100B, which is a calcium-binding protein found in astrocytes that has a number of studies in both experimental and human models [193,194]. From this perspective, it is a hard task to summarize all the most prominent biomarkers and those commonly found in animals and clinical studies. Despite that, it is possible to highlight a few of them, grouping them in three major categories: trophic factors, enzymes and epigenetic markers.

The first category involves both neurotrophic and gliotrophic factors. S100B is present in physiological conditions in the CNS due to its gliotrophic and neurotrophic roles. Initially, it was discovered that this marker plays a fundamental role in the differentiation and development of astrocytes but also in the neurite outgrowth. However, as well as the dual face of glial scar, the S100B overexpression also possess two contradictory activities: On one hand, its overexpression is often associated with injury events, such as spinal cord injury, brain traumas and stroke, and it displays a deleterious activity [195,196]. On the other hand, this protein also negatively modulates the neuroinflammation by the TNF-α pathway and other pro-inflammatory mediators and also the reduction of microgliosis, depending on the S100B extracellular concentration [196,197,198]. Insulin-like growth factor (IGF) has several biological activities in the CNS related to the brain development and the synaptoplasticity. Corne et al. [199] have detected reduced levels of IGF-1 and IGF-2 in the early chronic phase of TBI and an upregulation in the acute phase after TBI, indicating that the IGF system is differentially deregulated in the both acute and early chronic stages of TBI.

Glial fibrillary acidic protein (GFAP), a constitutive protein associated with astroglial damage and released after injury-induced impairment of the astroglial cytoskeleton, emerges as a biomarker following TBI, and it has been suggested that it may serve as a marker of focal lesions [200]. In addition, the protease ubiquitin C-terminal hydrolase-L1 (UCH-L1) has also been investigated as a biomarker following TBI. In an observational study, Diaz-Arrastia et al. [201] showed a relationship between GFAP and UCH-L1 markers, providing an indication that an analysis of both biomarkers together would be more effective than an analysis of each alone for the diagnosis and prognosis of TBI.

Although the CNS presents a high density of cell bodies, the extracellular matrix is present and composed mainly by glycosaminoglycans and proteoglycans, performing the classical roles of extracellular matrix but also providing a suitable microenvironment for BBB maintenance, neuroplasticity, synaptic transmission and microglial activity [202]. From this perspective, considering the morphological alteration triggered by TBI, would there be a biochemical and morphological alteration on the matrix? In order to shed some light on this question, Minta et al. [203] have investigated the behavior of matrix metalloproteinases (MMPs) after TBI, and have showed an increase of MMP-1, MMP-3 and MMP-10 in TBI patients, while MMP-2, MMP-9 and MMP-12 did not differ between both TBI and control patients. Such results indicate a differential role in the pathophysiology following human TBI.

The third category regards the epigenetic mechanisms involved in TBI events. Epigenetic markers are involved in gene expression mechanisms, such as histone methylation and miRNA, that modulate the status of regulation of genes by increasing or decreasing the susceptibility to translation processes [204]. Particularly in stroke events, some miRNA are enrolled in inflammatory functions, such as miR-424, which lead to microglial activation inhibition, and miR-155, which is associated with the TNF-α pathway [205]. These are only two examples from a broad range of miRNA that have already been associated with stroke events; however, most of them are also found in cardiovascular and metabolic diseases, such as arterial hypertension and diabetes [206].

## 3. Inflammasomes in the Context of Neuroinflammation and TBI Pathophysiology

As stated in Section 1.3 of the present review, neuroinflammation plays a critical role in the pathophysiology of TBI. In this context, in recent years, research has shed light on the role of inflammasomes in mediating the inflammatory process following TBI. Inflammasomes are multiprotein complexes that regulate the activation of pro-inflammatory cytokines, including IL-1β and IL-18. Understanding its involvement in neuroinflammation after TBI may provide valuable insights into the development of novel therapeutic approaches for mitigating the secondary damage caused by inflammatory responses following TBI.

Inflammasomes are cytosolic protein complexes present in innate immune cells, including microglia and macrophages, as well as in some non-immune cells like neurons and astrocytes [207]. Their primary function is to detect pathogen-associated molecular patterns (PAMPs) and damage-associated molecular patterns (DAMPs) [207]. Upon activation, inflammasome complexes are involved in the activation of caspase 1, which cleaves and activates pro-inflammatory cytokines, including IL-1β and IL-18 [208], which are potent mediators of inflammation.

In the context of TBI, DAMPs released from injured neurons, glial cells, and the vascular endothelium, such as ATP, ROS, and high-mobility group box 1 (HMGB1), can activate inflammasomes [209,210]. The best-characterized inflammasome is the NLRP3 (NOD-like receptor family, pyrin domain-containing 3) inflammasome [211]. Once activated, the NLRP3 inflammasome recruits the adapter protein ASC (apoptosis-associated speck-like protein containing a CARD), which in turn recruits and activates caspase 1 [212]. Caspase 1 then cleaves pro-IL-1β and pro-IL-18 into their active forms of IL-1β and IL-18, which are released into the extracellular space.

In addition to cytokine secretion, inflammasomes also trigger a form of cell death called pyroptosis [213]. Pyroptosis is an inflammatory form of programmed cell death characterized by cell swelling, plasma membrane rupture, and the release of pro-inflammatory intracellular contents [213]. This mechanism ultimately leads to the amplification of the inflammatory response and further exacerbates neuroinflammation.

The sustained activation of inflammasomes and the subsequent release of pro-inflammatory cytokines and pyroptosis can contribute to the progression of secondary injury after TBI [214]. The inflammatory cascade disrupts the BBB, exacerbates edema formation, promotes excitotoxicity, and leads to neuronal death and axonal injury [76]. These processes can ultimately worsen neurological outcomes and contribute to the development of long-term cognitive and motor deficits in TBI patients.

Given the pivotal role of inflammasomes in TBI-induced neuroinflammation, targeting inflammasomes has emerged as a potential therapeutic strategy [211,215]. Inhibiting inflammasome activation or blocking specific cytokines (e.g., IL-1β) has shown promising results in preclinical studies, ameliorating the extent of tissue damage and improving neurological outcomes [216]. However, further research is needed to identify safe and effective strategies for modulating inflammasome activity without compromising the necessary immune responses for brain repair and recovery.

Inflammasomes’ activation following TBI contributes to the amplification of inflammation, leading to secondary injury and neurological deficits. A proper understanding of the signaling pathways of inflammasomes in TBI pathophysiology opens new avenues for developing targeted therapeutic interventions to improve patient outcomes and promote neuroprotection after traumatic brain injury.

## 4. Translational Approach of Data Obtained in Animal Models of TBI and Limitations

Studies in animal models have provided a significant advance in the understanding of the pathophysiological aspects underlying TBI [217], allowing a preclinical evaluation of several therapeutic agents [218]. However, it is important to bear in mind the limitations in translating the findings arisen in animal models of TBI to clinical applications.

The complexity of TBI and the multitude of interacting pathways make it challenging to pinpoint specific therapeutic targets. Moreover, as pointed out, the majority of research on these signaling pathways has been conducted in preclinical models [219]. Such difficulty in clinical translation points out the importance of re-examining the present status of animal models of TBI [220]. One of the biggest challenges in the translation process is related to both the anatomical and physiological differences between rodent and human brains [221] and how models of impact on human TBI can be simulated in animal models [219,222]. The use of large animals, such as sheep, rabbits, pigs, and non-human primates has allowed a more complete understanding concerning therapeutic targets following TBI in human beings [218,223].

Notwithstanding, as stressed above, TBI animal models have inherent limitations due to species differences and the simplified replication of complex injury mechanisms, hindering direct translation to the clinical setting, since models often focus on controlled injury paradigms, while real-world TBI patients exhibit considerable heterogeneity. In light of this, further studies are needed to validate their relevance in human TBI.

Also, given the heterogeneous nature and complex pathophysiology of TBI, interventions that solely target isolated approaches, such as specific metabolic, signalizing, inflammatory or apoptotic pathways, may not fully contemplate the diverse and interconnected pathophysiological processes involved in TBI [224]. Efforts should be made to incorporate diverse patient characteristics and injury profiles to better reflect the clinical population, since methods for the diagnosis and classification of patients suffering from TBI have been considered insufficient to allow the effectivity of current and new therapeutic approaches [225]. Thus, the heterogeneity of TBI patients, including variations in injury severity, age, and comorbidities, challenges the development of targeted therapies that can be universally effective. In light of this, the characterization of biomarkers associated with distinct steps of TBI progression will contribute to a better approaching concerning adequate treatments in TBI sufferers in order to optimize trial planning, medical decision making, and improve individualized and targeted therapeutic interventions [226].

## 5. Conclusions

In this review, we have highlighted the main elements underlying both the primary and secondary injuries following TBI. Primary injury can be elicited either by penetrating (open-head) or nonpenetrating (closed-head or blunt) lesions, both causing mechanical tissue deformation, disturbance in the blood flow, osmotic imbalance, activation of inflammatory cells, and cell death. Secondary injury, in turn, involves a cascade of complex pathophysiological processes that can exacerbate the initial damage and lead to further neurological dysfunction and tissue loss.

Brain edema, inflammatory response, oxidative stress, metabolic disturbances, and oxidative stress are crucial elements following TBI, all of them contributing to the tissue damage and cognitive impairment.

In light of the above, efforts to mitigate secondary brain injury aim to prevent or attenuate the deleterious processes and optimize conditions for neuronal recovery and repair. Understanding and targeting secondary brain injury is of paramount importance in the management of TBI, as early recognition and interventions to reduce secondary injury can potentially improve patient outcomes and promote neurological recovery.

## Figures and Tables

**Table 1:** Examples of studies employing animal models explored in the present review.

Experimental Model	Animal Model	Type of Injury	Pathway/Structure Evaluated	Outcomes	Reference
Marmarou weight drop model	Rat	Focal	Apoptotic pathways	Cyto c released, activating caspase 3 apoptotic pathway	Buki et al. [23]
Weight drop TBI model	Rat	Focal	Oxidative stress, Inflammation	Oxidative injury, blood-brain barrier disruption, microglial activation	Choi et al. [24]
Controlled cortical impact injury model	Rat	Focal	Energetic metabolism	Mitochondrial dysfunction	Xiong et al. [25]
Controlled cortical impact injury model	Rat	Focal	Excitotoxicity	Excitotoxic injury caused by higher concentrations of aspartate and glutamate in the brain parenchyma	Palmer et al. [19]
Controlled cortical impact model	Mouse	Focal	Microglia	Microglial activation, synaptic loss	Krukowski et al. [26]
Closed-head injury model	Mouse	Diffuse	Proinflammatory pathways	Increase in Interleukin 1-expression following repetitive brain lesion	Wu et al. [27]
Marmarou weight drop model	Rat	Diffuse	Structural organization	Axonal injury, brain edema	Foda and Marmarou [28]
Marmarou weight drop model		Focal		Altered axolemmal permeability, cytoskeletal disturbances	Povlishock et al. [29]
Dorsal column crush injury model	Rat	Focal/diffuse	Tissue swelling (brain edema)	Water channel aquaporin-4 cell surface increases in response to hypoxia-induced cell swelling	Kitchen et al. [30]
Marmarou weight drop model	Rat	Focal	Inflammatory response	Proliferation and increase of reactive astrocytes and microglia	Bye et al. [31]
Central fluid percussion injury mode	Mouse	Diffuse	Structural organization	Neurofilament phosphorylation, myelin impairment	Ozen et al. [32]
Maryland closed-head injury model	Rat	Diffuse	Axonal structure, apoptotic pathways	Petechial hemorrhage, axonal damage, caspase 3 activation	Kilbourne et al. [33]
Closed-head impact model of engineered rotational acceleration	Mouse	Diffuse	Axonal structure, glial structure	White matter gliosis, axonal damage	Bashir et al. [34]
Blast TBI model	Rat	Diffuse	Axonal structure	Axonal injury	Zhang et al. [35]
Repetitive blast TBI model	Mouse	Diffuse	Structural organization, glial structure	Tau phosphorylation, microglial activation	Bugay et al. [36]

## Data Availability

All data are available within the article.
